# Cortical morphological alterations in vestibular migraine: insights from surface-based morphometry and machine learning

**DOI:** 10.1186/s10194-025-02232-8

**Published:** 2025-12-01

**Authors:** Wen Chen, Hongru Zhao, Xing Xiong, Qifang Feng, Lingling Dai, Jun Ke, Chunhong Hu

**Affiliations:** 1 Department of Radiology, The First Affiliated Hospital of Soochow University, Shizi Street 188, Suzhou, Jiangsu 215006 P. R. China; 2 Institute of Medical Imaging, Soochow University, Soochow, Jiangsu Province P. R. China; 3 Department of Neurology, The First Affiliated Hospital of Soochow University, Suzhou, Jiangsu 215006 China

**Keywords:** Vestibular migraine, Magnetic resonance imaging, Surface-based morphometry, Cortical thickness, Surface area, Machine learning

## Abstract

**Background:**

Previous surface-based morphometry (SBM) research on cortical morphology in vestibular migraine (VM) has been limited by a small sample size. This study aims to validate cortical morphological alterations in VM using SBM and explore their clinical implications with a larger sample.

**Methods:**

Fifty-five patients with VM and 65 healthy controls (HCs) underwent structural T1-weighted MRI. Cortical morphological features, including cortical thickness, curvature, surface area, and local gyrification index, were assessed using SBM with FreeSurfer. Statistical analyses were conducted to examine inter-group differences and correlations between cortical alterations and clinical features. A linear support vector machine (SVM) classifier was applied to evaluate the performance of cortical morphological differences in distinguishing VM patients from HCs.

**Results:**

The SBM analysis revealed significant cortical differences between the VM group and HCs. Specifically, cortical thickness was significantly reduced in the right superior frontal gyrus, superior parietal lobule, and precuneus in the VM group as compared to HCs. Additionally, surface area was significantly smaller in the right rostral middle frontal cortex in the VM cohort. No significant correlations were found between the cortical morphological features and any clinical indicators. The SVM classification model achieved moderate efficacy (area under the curve = 0.775, *p* < 0.001) in distinguishing VM patients from HCs.

**Conclusions:**

These findings demonstrate significant cortical morphological alterations in the frontoparietal regions of patients with VM, which may be associated with dizziness, pain, and emotional and cognitive dysfunctions. The identified cortical differences have the potential to serve as neuroimaging markers for VM.

## Introduction

Vestibular migraine (VM) is a prevalent neurological disorder characterized by headache and recurrent episodes of vertigo, along with nausea, vomiting, and balance disturbances [1, 2]. It affects 1%–3% of the general population [3, 4] and is the leading cause of episodic vertigo, as well as the second most common vestibular syndrome etiology [4, 5]. Despite its significant impact on quality of life and the substantial economic burden it poses on both individuals and society [4, 5], VM is frequently underdiagnosed, and its underlying pathophysiology remains poorly understood [6, 7]. Consequently, investigating the neural mechanisms behind VM and identifying reliable biomarkers are crucial steps toward improving diagnostic accuracy and therapeutic outcomes for affected individuals.

The development of neuroimaging techniques has significantly enhanced our understanding of the neural mechanisms underlying VM, with voxel-based morphometry (VBM) becoming the dominant method in structural research. Existing VBM-based evidence links VM to structural alterations in brain regions involved in vestibular processing, pain modulation, and multisensory integration [8–11]. However, despite providing valuable insights, VBM has several limitations, such as its mixed assessment of gray matter (GM) that encompasses cortical thickness, folding, and surface area. It is also less resistant to noise and mis-segmentation, and its accuracy is hindered by the limited resolution of the voxel grid and partial volume effects at the boundaries of complex structures [12]. To address these issues, surface-based morphometry (SBM) has emerged as a more specific and precise method for assessing cortical alterations. SBM not only offers more detailed information about cortical structural alterations (i.e., cortical thickness, folding, and surface area), but it has also been shown to be more sensitive and accurate than VBM in detecting GM atrophy [13].

Indeed, there has been one SBM study investigating cortical morphology in VM patients [14]. It identified cortical thinning in the bilateral inferior temporal gyrus, left middle temporal gyrus, and right superior parietal lobule (SPL), alongside reduced sulcus depth in parietal regions. These observations point to morphological disruptions in areas involved in multisensory integration, which may be linked to VM symptoms and diminished life quality. However, the study involved a relatively small sample size of patients and healthy controls (HCs), which could impact the generalizability and statistical power of the findings. Additional research with a larger sample is needed to validate the reproducibility of cortical alterations in VM and their connection to clinical features. This is an important and worthwhile area of research, as numerous VBM studies on VM have produced highly heterogeneous and conflicting results [8–11], likely due to small sample sizes and inconsistencies in clinical profiles across studies. Given these discrepancies in VBM research and the clinical heterogeneity of VM [15–17], we hypothesize that SBM findings in VM may also demonstrate similar variability.

The aim of this study was to test our hypothesis by validating cortical morphological alterations in VM using the SBM method and exploring their clinical associations in a relatively larger sample. By increasing the sample size, we sought to enhance statistical robustness, confirm the reproducibility of previous findings, and potentially reveal new cortical regions involved in VM pathophysiology. Moreover, given the growing importance of machine learning in neuroimaging biomarker identification [18], we applied support vector machine (SVM) analysis [19] to test whether cortical features could differentiate VM patients from HCs. SVM was chosen for its ability to perform robustly with small sample sizes [20], its straightforward interpretability of feature weights [21], and its widespread adoption in neuroimaging research and recognition as a benchmark method [22, 23]. Given the preliminary nature of this exploratory analysis, only the SVM model was employed to maintain methodological simplicity. Compared to prior research, the novel incorporation of SVM-based classification in this work may aid in identifying potential neuroimaging markers and their possible clinical translation.

## Materials and methods

### Participants

This study received approval from the Ethics Committee of the First Affiliated Hospital of Soochow University (Approval no. 2021246). All participants provided written informed consent prior to their inclusion in the research. A total of 55 right-handed patients with VM from the vertigo and migraine outpatient center of our hospital were included in this study. All the patients were diagnosed by the same experienced neurologist (H.R.Z) according to the criteria published by the Bárány Society and International Headache Society (ICHD-3 beta, appendix) [24, 25]. To rule out peripheral vestibular disorders, a series of comprehensive evaluations were conducted, including videonystagmography, vestibular caloric test, video head impulse test, and audiometry tests. Demographic and clinical information was gathered from all participants using a standardized questionnaire. The collected data encompassed variables such as age, sex, education level, duration of migraine and vertigo, headache frequency (measured in days per month), scores on the 10-point Visual Analog Scale (VAS), Dizziness Handicap Inventory (DHI), Migraine Disability Assessment Scale (MIDAS), Headache Impact Test-6 (HIT-6), Patient Health Questionnaire-9 (PHQ-9), and Generalized Anxiety Disorder-7 (GAD-7). All patients were in the interictal phase, defined as being free of both migraine and vertigo attacks for at least three days before and one day after the MRI scan. None of the patients fulfilled the diagnostic criteria for chronic migraine according to the International Classification of Headache Disorders, 3rd edition (ICHD-3) [24]. Moreover, no patients had used any prophylactic medication, nor were they undergoing treatment at the time of the study. Some patients (*n* = 29) had previously used therapeutic medications but had refrained from taking any within the three days prior to the MRI scan to minimize potential confounding effects.

In addition, 65 HCs balanced for age, sex, and education level were included. These controls had no personal or familial history of clinically diagnosed vestibular disorders, migraine, or other primary headache disorders, as per established diagnostic guidelines. The exclusion criteria applied to both the patient and control groups comprised left-handedness, other neurological or mental disorders, other pain conditions, drug or alcohol abuse, and contraindications to MRI.

### MRI acquisition

An MRI scan was performed on all participants using a 3.0-Tesla machine (MAGNETOM Skyra, Siemens Healthcare, Erlangen, Germany) with a 16-channel head and neck joint coil. Foam padding was applied to minimize head movement, and earplugs were provided to reduce scanning noise. Participants were instructed to lie still in a supine position with their eyes closed, staying awake and relaxed throughout the scan, and were explicitly reminded to avoid any head movement. High-resolution T1-weighted anatomical images were acquired using a sagittal fast spoiled gradient recalled echo sequence with the following parameters: repetition time = 2300 ms, echo time = 2.98 ms, field of view = 256 × 256 mm^2^, matrix = 256 × 256, slice thickness = 1 mm, voxel size = 1 × 1 × 1 mm^3^, and 192 slices. After acquisition, the images were promptly reviewed by two experienced radiologists to confirm the absence of overt structural abnormalities. Participants whose images showed visible motion artifacts or poor image quality would be excluded from further analysis, and all participants included in this study satisfied the quality control criteria.

### Data processing

Structural MRI data in Digital Imaging and Communications in Medicine format were initially converted to Neuroimaging Informatics Technology Initiative files. Cortical SBM analyses were conducted using FreeSurfer (version 7.4.1, http://surfer.nmr.mgh.harvard.edu/) [26, 27], an advanced software suite for automated cortical surface reconstruction and normalization. The data of each participant underwent approximately 20 h of preprocessing, which encompassed motion correction, skull stripping, transformation to Talairach space, segmentation of gray and white matter tissues, intensity normalization, tessellation of the gray–white matter boundaries, automated topology correction, and surface deformation. All images underwent visual inspection to identify potential reconstruction issues, such as skull-stripping failures, substantial segmentation defects, or inaccuracies in the white-matter and pial surface reconstruction. Detected surface inaccuracies were manually corrected using FreeSurfer’s editing utilities, after which the images were reprocessed through the FreeSurfer pipeline. This iterative procedure was repeated until all surface errors were resolved.

Following preprocessing, cortical thickness, curvature, surface area, and local gyrification index were computed and subsequently registered to the fsaverage standard space. The local gyrification index was smoothed with a 5-mm full-width at half maximum (FWHM) Gaussian kernel, while other metrics (i.e., cortical thickness, curvature, and surface area) were smoothed using a 10-mm FWHM Gaussian kernel.

### Statistical analyses

Group differences in cortical morphological characteristics between VM patients and HCs were assessed using vertex-wise two-sample t-tests on metrics including cortical thickness, curvature, surface area, and local gyrification index. Analyses were performed in the BrainStat toolbox (http://brainstat.readthedocs.io/en/master/) [28], with age, sex, and education level as covariates to adjust for potential confounds. Statistical significance was determined using random field theory for multiple comparisons correction across the whole cortex, with a vertex-level threshold of *p* < 0.001 and a cluster-level threshold of *p* < 0.05. Surviving clusters were labeled according to the Desikan-Killiany atlas and reported with their cluster sizes, peak t values, and peak coordinates in Montreal Neurologic Institute space.

To further explore the clinical relevance of cortical morphological alterations in VM patients, the mean metric values within each significant cluster were extracted for each individual in the VM cohort. Partial correlation analyses were conducted using R software (version 4.5.1; R Foundation for Statistical Computing, Vienna, Austria), controlling for age, sex, and education level, to examine the relationships between these cortical morphological measures and clinical variables. Statistical significance was set at *p* < 0.05 after false discovery rate (FDR) correction, i.e., at an FDR-corrected *p* (*p*_FDR_) < 0.05.

Demographic and clinical data were analyzed using SPSS software (version 27.0.1; IBM Corp., Armonk, NY, USA). For continuous variables, the normality of the data was first evaluated using the Shapiro–Wilk test. Then, group differences between VM patients and HCs were assessed using two-sample t-tests for normally distributed data or Mann–Whitney U tests for non-normally distributed data. For categorical variables, chi-square tests or Fisher’s exact tests were used when appropriate. Statistical significance was set at *p* < 0.05.

### Machine learning classification analysis

To further determine whether the cortical morphological features could serve as potential neuroimaging markers for distinguishing VM patients from HCs, SVM classification analysis was performed using the LIBSVM toolbox (http://www.csie.ntu.edu.tw/~cjlin/libsvm/) [29]. The classifier was trained using a combination of the identified cortical morphological characteristics showing significant inter-group differences. Participants were labeled as 1 for those with VM and −1 for HCs. Before model fitting, all features were normalized to the range of 0–1 using min–max scaling, with scaling parameters derived from the training data and subsequently applied to the test data. The regularization parameter *C* was optimized using a grid *C* = 2^*k*^ with *k* ranging from −10 to 10 in increments of 0.2. A linear kernel SVM was then implemented to perform the classifier training, which allows straightforward interpretation of the feature weights and minimizes the risk of overfitting [30]. In this model, the classifier constructs a separating hyperplane which maximizes the margin between the hyperplane and the support vectors [30]. The decision function can be expressed as:

$$f\left( x \right) = {w_1}{x_1} + {w_2}{x_2} + {\text{ }} \ldots {\text{ }} + {w_i}{x_i} + b,$$

where *x*_i_ represents the *i*th feature vector, *w*_i_ denotes the corresponding feature weight, *b* is the bias, and *f*(*x*) is the decision value. A participant is classified as VM if *f*(*x*) > 0, and as control if *f*(*x*) < 0. Model evaluation was performed using a leave-one-out cross-validation approach, and the classifier’s performance was primarily assessed using the area under the receiver operating characteristic curve (AUC), along with accuracy, sensitivity, and specificity.

### Validation analyses

To assess the robustness of our results, two validation analyses were conducted. (1) A non-parametric permutation test with 1,000 permutations was applied to the SVM classification to assess the significance of both AUC and accuracy. (2) The SVM classification was further validated using a nested 5-fold cross-validation approach. In this framework, the outer 5-fold loop evaluated model performance, while the inner 5-fold loop optimized the hyperparameter *C*. Stratified sampling was employed to ensure balanced class distribution within each fold. For model performance metrics, accuracy was averaged across the outer folds, whereas AUC, sensitivity, and specificity were computed from the pooled outer-test predictions. Linear kernel weights were averaged across the outer folds.

## Results

### Demographic and clinical characteristics

Table 1 summarizes the demographic and clinical characteristics of the study cohort. There were no significant differences in age (*p* = 0.133), sex (*p* = 0.566), or education level (*p* = 0.253) between VM patients and HCs.

**Table 1:** Demographic and clinical characteristics

Items	VM group (*n* = 55)	HCs (*n* = 65)	*p*-value
Age (years)	45.05 ± 11.65	47.80 ± 13.11	0.133^a^
Sex (male/female)	8/47	12/53	0.566^b^
Education level (years)	10.60 ± 4.89	11.83 ± 4.81	0.253^a^
Migraine disease duration (years)	12.17 ± 11.47	−	−
Headache occurrence (yes/no)	51/4	−	−
Headache frequency/month	2.21 ± 2.32	−	−
Headache laterality (bilateral/left/right/variable)	30/8/10/3	−	−
Vertigo disease duration (years)	7.14 ± 8.78	−	−
History of therapeutic medication use (yes/no)	29/26	−	−
VAS	6.36 ± 2.16	−	−
DHI	51.40 ± 17.13	−	−
MIDAS	10.61 ± 11.55	−	−
HIT-6	55.07 ± 10.10	−	−
PHQ-9	5.76 ± 5.13	−	−
GAD-7	4.60 ± 4.03	−	−

Data are presented as mean ± standard deviation, unless otherwise indicated. Headache laterality was assessed only among the 51 patients with headache

VM = vestibular migraine; HCs = healthy controls; *n* = number of subjects; VAS = Visual Analog Scale; DHI = Dizziness Handicap Inventory; MIDAS = Migraine Disability Assessment Scale; HIT-6 = Headache Impact Test-6; PHQ-9 = Patient Health Questionnaire-9; GAD-7 = Generalized Anxiety Disorder-7

^a^ *p*-value with Mann–Whitney U test

^b^ *p*-value with chi-square test

### SBM results

**Fig. 1:**
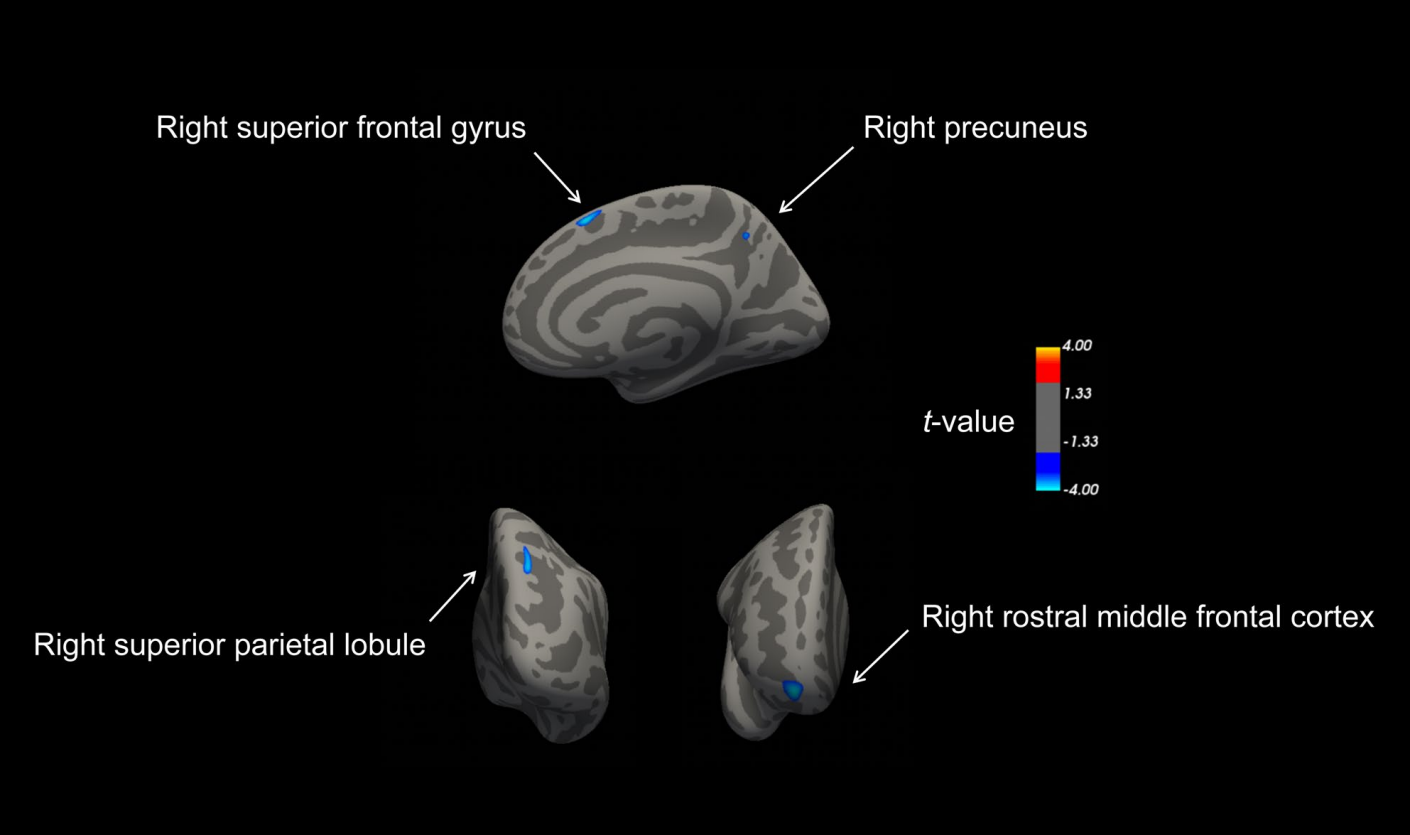
Illustration of brain regions with significantly altered cortical morphology in VM patients relative to HCs. Cortical thickness was reduced in the right superior frontal gyrus, superior parietal lobule, and precuneus in VM patients. Additionally, surface area was smaller in the right rostral middle frontal cortex. The color bar represents the t-value from the two-sample t-test between the VM group and HCs. A positive t-value indicates higher values in the VM group compared to HCs, while a negative t-value indicates lower values in the VM group. Statistical significance was determined using random field theory correction, with a vertex-level threshold of *p* < 0.001 and a cluster-level threshold of *p* < 0.05. VM = vestibular migraine; HCs = healthy controls

The SBM analysis revealed several cortical differences between the VM group and HCs after controlling for age, sex, and education level. Specifically, cortical thickness was significantly lower in the right superior frontal gyrus (SFG), SPL, and precuneus in the VM group as compared to HCs (vertex *p* < 0.001, cluster *p* < 0.05, random field theory corrected). Furthermore, surface area was significantly reduced in the right rostral middle frontal cortex in the VM cohort (vertex *p* < 0.001, cluster *p* < 0.05, random field theory corrected). No regions exhibited significant differences in cortical curvature or local gyrification index between the two groups. A detailed presentation of the results can be found in Table 2; Fig. 1.

**Table 2:** Brain regions with significantly altered cortical morphology in VM patients compared to HCs (vertex *p* < 0.001, cluster *p* < 0.05, random field theory corrected)

Brain regions / conditions	Cluster size (mm^2^)	Peak MNI coordinates			Peak t-value
		X	Y	Z	
*Cortical thickness*:					
VM group < HCs					
Right superior frontal gyrus	90.83	7.4	18	56.1	−3.880
Right superior parietal lobule	127.59	21.4	−67.7	38.8	−3.765
Right precuneus	23.33	9.6	−54.5	44.2	−3.556
*Surface area*:					
VM group < HCs					
Right rostral middle frontal cortex	184.23	17.4	58.7	−12.7	−3.991

VM = vestibular migraine; HCs = healthy controls; MNI = Montreal Neurologic Institute

### Clinical relevance

In the VM cohort, after controlling for age, sex, and education level, no significant correlations were found between the cortical morphological features and any clinical indicators, including migraine disease duration, headache occurrence, headache frequency, vertigo disease duration, history of therapeutic medication use, VAS, DHI, MIDAS, HIT-6, PHQ-9, and GAD-7 (all *p*_FDR_ >0.05).

### Machine learning classification results

**Fig. 2:**
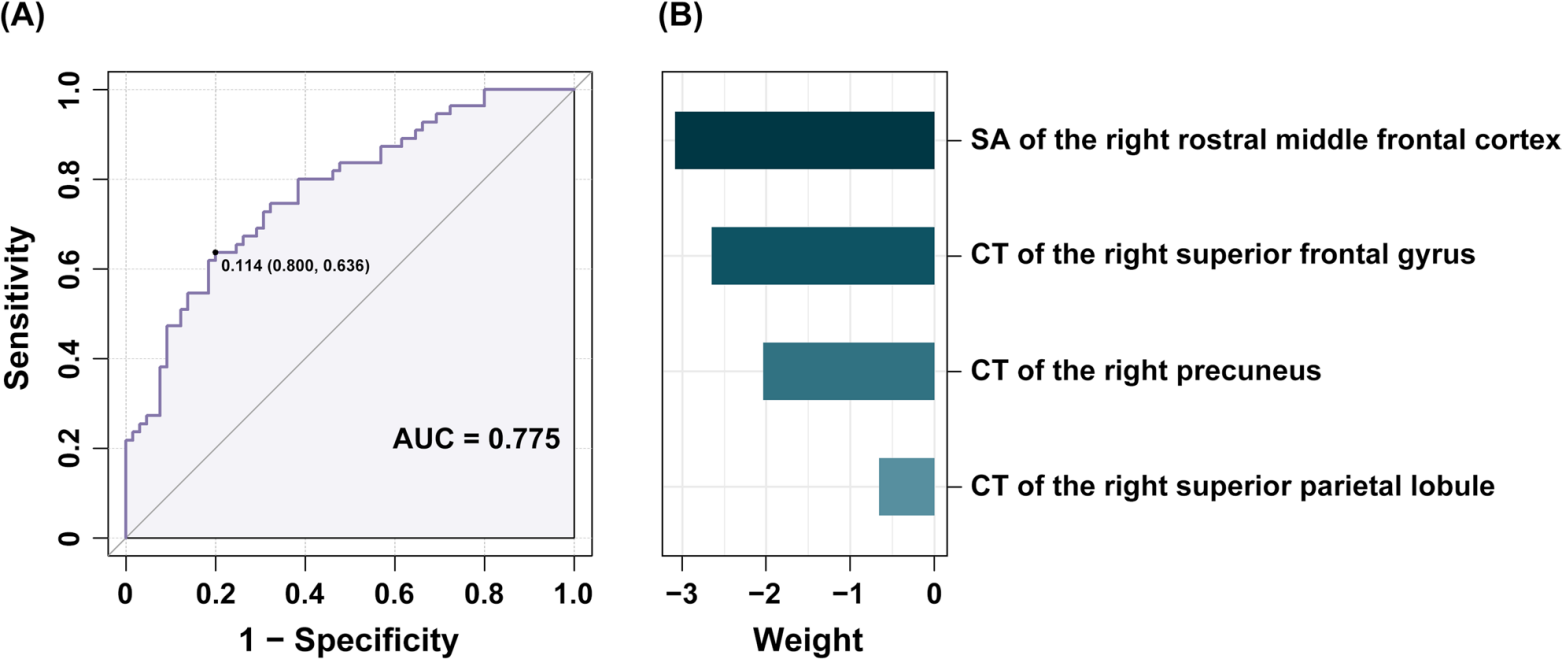
SVM classification results. (**A**) The receiver operating characteristic curve indicates that the SVM model achieved moderate performance in distinguishing VM patients from HCs, with an AUC of 0.775 (*p* < 0.001). The black dot on the curve represents the optimal cutoff point, and the adjacent values denote the cutoff threshold and the corresponding specificity and sensitivity. (**B**) The bar plot shows the feature weights in the model. The SA of the right rostral middle frontal cortex and the CT of the right superior frontal gyrus, precuneus, and superior parietal lobule had weights of −3.090, −2.648, −2.035, and −0.659, respectively. All identified features exhibited negative weights, indicating that these features had a negative influence on the decision function, i.e., lower values of these cortical features contributed to classifying participants as having VM, whereas higher values contributed to classifying them as HCs. SVM = support vector machine; VM = vestibular migraine; HCs = healthy controls; AUC = area under the receiver operating characteristic curve; SA = surface area; CT = cortical thickness

Our SVM classification model demonstrated moderate performance in distinguishing VM patients from HCs, achieving an AUC of 0.775 (*p* < 0.001). The model’s accuracy, sensitivity, and specificity were 69.17%, 63.64%, and 80.00%, respectively. In the model, the cortical thickness of the right superior frontal gyrus, right superior parietal lobule, and right precuneus had weights of −2.648, −0.659, and −2.035, respectively. The surface area of the right rostral middle frontal cortex had a weight of −3.090. Detailed information regarding the machine learning classification results is provided in Fig. 2.

### Validation results

The validation analyses supported the robustness of our findings within the current dataset. (1) Permutation testing confirmed the reliability of the classifier, with both the AUC (*p* < 0.001) and accuracy (*p* = 0.001) reaching statistically significant. (2) In the nested 5-fold cross-validation, the SVM achieved an average classification accuracy of 71.67%, with an AUC of 0.756, a sensitivity of 65.45%, and a specificity of 76.92%. The cortical thickness of the right superior frontal gyrus, right superior parietal lobule, and right precuneus, as well as the surface area of the right rostral middle frontal cortex, had average weights of −2.167, −0.935, −1.478, and −2.471, respectively. These findings from the nested 5-fold cross-validation were highly consistent with the main results obtained using leave-one-out cross-validation.

## Discussion

The present study investigated cortical morphological alterations in patients with VM using a combined SBM and machine learning approach. Our study revealed two major findings: (1) Patients with VM showed significant cortical thinning in the right superior frontal, superior parietal, and precuneus regions, and reduced surface area in the right rostral middle frontal cortex; (2) The SVM classification model achieved moderate efficacy (AUC = 0.775, *p* < 0.001) in distinguishing VM patients from HCs. These results reveal specific cortical morphological abnormalities that may serve as potential neuroanatomical markers of VM, providing structural neuroimaging evidence for frontoparietal involvement in its pathophysiology.

The SFG, a part of the prefrontal cortex (PFC), has been identified within the vestibular system, contributing to ocular motor control and nystagmus processing [31, 32]. It also integrates somatosensory and vestibular information [33], essential for maintaining spatial orientation and balance. Additionally, the SFG participates in pain modulation [34] and emotion regulation [35]. Both functional [14, 36–38] and structural [10, 39] alterations in the SFG have been reported in VM. Taken together, cortical thinning in the SFG may underlie the multifaceted dysfunctions of VM—encompassing impaired vestibular processing, altered pain regulation, disrupted multisensory integration, and emotional dysregulation—that collectively contribute to the clinical symptomatology of the disorder. However, the reduction in cortical thickness observed in the SFG in this study should be interpreted with caution, as the cluster size (90.83 mm^2^) is relatively small, which may limit the reliability of this finding. Further validation with larger samples and more refined imaging techniques is warranted to confirm the robustness of the result. The rostral middle frontal cortex, also a component of the PFC, is implicated in pain regulation and the cognitive processing of pain experiences [40]. Therefore, the observed reduction in surface area in this region may be related to disruptions in the pain processing networks involved with VM. Notably, no overlap was observed in the alterations between these two regions, i.e., the SFG showed a reduction in cortical thickness, while the rostral middle frontal cortex exhibited a decrease in surface area in VM. This suggests that distinct cortical morphological metrics capture different structural alterations, emphasizing the complementary nature of these measurements in providing a more comprehensive characterization of cortical alterations.

Another important finding of the present study is the cortical thinning observed in the right SPL in patients with VM. This result aligns with previous neuroimaging studies that have reported SPL alterations in VM [9, 14, 37, 41]. In particular, a recent SBM study by Zhe et al. [14] also observed a reduction in cortical thickness in the right SPL, a finding that is fully consistent with ours. This convergence across studies suggests that reduced cortical thickness in the right SPL may represent a relatively stable and reproducible neuroimaging feature associated with VM. The SPL is a critical component of the parietofrontal network, involved in the perceptual matrix of pain [42]. It also contains major parts of the sensory cortex responsible for spatial orientation as well as the processing and interpretation of sensory information [43]. These functional attributes imply that the observed cortical thinning in the SPL may be associated with disruptions in pain processing and spatial orientation, both of which are relevant to the clinical manifestations of VM. This inference is further substantiated by reports of SPL abnormalities in patients with chronic migraine [44], cluster headache [45], medication overuse headache [46], and chronic unilateral vestibulopathy [47], which collectively support SPL’s wider role in pain and sensory processing dysfunctions.

Our study also identified significant cortical thinning in the precuneus of VM patients. The precuneus is a critical node in the default mode network, which is involved in self-referential processing and various high-level cognitive functions such as episodic memory and consciousness [48, 49]. In the vestibular domain, it has been implicated in integrating visual and vestibular information, as well as spatial positioning and perception [50–52]. Therefore, structural degradation in this region might be related to the deficits in balance, coordination, and motor behavior experienced by VM patients [50–52]. Moreover, the precuneus is recognized for its involvement in pain sensitivity [53, 54]. Goffaux et al. [55] reported that pain-evoked responses in the contralateral precuneus were closely associated with pain sensitivity among healthy adults. Additionally, Emerson et al. [56] observed a significant inverse relationship between pain sensitivity and GM density in the precuneus. As such, the reduced cortical thickness in precuneus observed in VM may also be linked to enhanced pain sensitivity underlying the heightened pain perception in these patients. However, given that the cluster identified in the precuneus was very small (23.33 mm^2^), this finding should be considered preliminary and interpreted with caution, and further studies with larger cohorts and higher spatial resolution are required to confirm the reproducibility of this observation.

It is noteworthy that, compared to the prior SBM analysis by Zhe et al. [14], we did not observe thinning in regions such as the inferior and middle temporal gyri, despite the aforementioned cortical thinning in the SPL. This discrepancy is consistent with the variability seen in VBM studies of VM [8–11], which report some non-overlapping brain regions. Such differences may result from variations in imaging techniques, analysis strategies, sample sizes, or from the inherent heterogeneity within the VM population, including differing proportions of patients with or without headache, variations in disease duration, and accompanying symptoms [2]. Besides, in our study, no significant correlations were found between cortical morphological features and clinical indicators. This may be attributed to several factors. First, the complex interplay of genetic, environmental, and disease-related influences on cortical morphology may obscure direct associations with clinical measures. Second, the subjective nature and limited sensitivity of the clinical scales used may have been insufficient to capture subtle structural alterations. Third, the heterogeneity of VM, in terms of symptom profile, disease duration, and severity, may further complicate the identification of consistent correlations. Finally, since our study focused on interictal patients, the absence of data during acute attacks could have masked potential relationships between clinical features and cortical morphology. Nonetheless, the cortical morphological alterations we observed are objective in nature and likely reflect disease-related, multifactorial structural characteristics of the brain. These findings provide objective evidence of neural involvement, offering valuable insights into VM pathophysiology beyond symptom manifestation. As for the deeper implications of these features—whether they are stable trait-like or state-related characteristics—they remain difficult to categorize. While scanning all patients during the interictal phase largely minimizes the influence of acute attack states, the current cross-sectional design makes it challenging to distinguish whether the observed morphological alterations are associated with the ongoing disease process, i.e., a broader disease state spanning the course of the disorder, or whether they reflect more stable trait-like characteristics independent of disease fluctuations. Some features may predate disease onset and serve as predisposing factors, whereas others may persist after symptom remission or treatment as long-term consequences of the disorder, both of which could be considered trait-like. Longitudinal or treatment follow-up studies that track changes over time would allow for distinguishing these possibilities.

Interestingly, all regions showing significant cortical alterations in our study were located in the right hemisphere. This right-lateralized pattern appears unlikely to be fully explained by headache laterality, as most patients in our sample experienced bilateral headaches. Given that all participants were right-handed, a more plausible—though still tentative—interpretation is that the observed pattern aligns with the hemispheric specialization typically seen in right-handed individuals. Accumulating evidence from functional neuroimaging studies has suggested a right-hemispheric bias in pain processing [57–59]. For instance, Symonds et al. [59] reported that activation in several cortical regions during acute pain stimulation was localized either exclusively to the right hemisphere or showed strong right-lateralization. Apart from pain processing, the vestibular system has also been recognized to exhibit right-hemispheric dominance in right-handed populations [60]. In addition, the right hemisphere is generally considered dominant in emotional processing and regulation [61]. These functional domains are related to the cortical regions identified in our study and are closely implicated in the pathophysiology of VM.

Currently, most neuroimaging research relies on traditional group-level statistical comparisons to draw conclusions. While informative, such approaches lack direct applicability to individual patients and thus offer limited translational value. In clinical practice, underdiagnosis of VM remains common [62], likely due to its poorly understood pathophysiological mechanisms and the absence of specific biomarkers [63]. The SVM is an analytical method which allows individual-level characterization, and therefore hold promise for clinical translation [19]. In the present study, we employed a linear SVM classifier using a combination of cortical morphological features to discriminate VM patients from HCs. The model achieved a moderate AUC of 0.775, suggesting that cortical morphological alterations may serve as potential neuroimaging markers for individualized differentiation between VM patients and HCs. However, given the modest classification performance, these features should be regarded as exploratory rather than conclusive biomarkers. To determine their reproducibility and generalizability, further validation using larger, independent, and multicenter datasets is required before these features can be considered clinically applicable. Nonetheless, the present findings suggest a preliminary translational potential of the SVM-based model, which may serve as a step toward developing imaging-based tools for the early and objective assessment of VM in clinical settings. Notably, the surface area of the right rostral middle frontal cortex had the largest absolute weight (|−3.090|), highlighting its contribution to the classification and the significance of this characteristic.

Overall, extending the work of Zhe et al. [14], the present study strengthened the statistical robustness of SBM analyses by including a larger cohort. Our findings partially replicated those of previous research [14], while also identifying new cortical regions that may be implicated in the pathophysiology of VM. Furthermore, the incorporation of SVM-based classification provided additional, data-driven evidence supporting the potential of cortical morphological features as candidate neuroimaging biomarkers for VM and underscored their possible relevance for future clinical translation.

The present study had several limitations. First, while the sample size was relatively larger compared to existing research, further studies involving a larger cohort is needed to confirm the current findings. Second, as all participants were recruited from a single center, the generalizability of our results should be assessed through validation with multicenter datasets. In this context, the proposed cortical morphological biomarkers remain at an early exploratory phase and require rigorous verification with independent samples collected across multiple institutions and MRI scanners. Third, the demographic characteristics of the HCs were only balanced rather than strictly matched to the patient group. Despite including these variables as covariates in the statistical analyses, potential confounding may not be completely ruled out, and future studies with more precisely matched HCs are warranted to confirm the present findings. Fourth, detailed finer clinical information—such as the prevalence and severity of both migraine and vestibular symptoms, the duration of moderate-to-severe vestibular episodes, the frequency of vestibular symptoms, the number of episodes involving both headache and vestibular symptoms, aura and its subtypes, as well as the specific types of prior therapeutic medications and their treatment durations—was not comprehensively collected for all patients. Future studies with more complete symptom-level data collection are necessary to strengthen the clinical characterization of VM and to better explore potential associations with cortical morphometric abnormalities. Fifth, it should be noted that the clusters identified in the SFG and especially the precuneus were small, and the interpretations of these results should therefore be made with caution. Additional studies using larger samples and improved spatial resolution are recommended to verify these observations. Sixth, our study did not include comparisons with other types of migraines, such as migraine without aura. Future studies that incorporate these comparisons would be valuable, offering deeper insights into both shared features across migraine subtypes and unique characteristics specific to each form. Last, the cross-sectional design of our study limits the ability to establish causal relationships between cortical morphological alterations and disease progression or treatment effects. Longitudinal prospective studies are needed to address these issues.

In conclusion, our study revealed significant cortical morphological alterations in the frontoparietal regions of patients with VM, which may be associated with dizziness, pain, as well as emotional and cognitive dysfunctions. These findings would enhance our current understanding of this disease from the perspective of cortical morphological abnormalities. The identified cortical differences could serve as potential neuroimaging markers of VM patients.

## Data Availability

The datasets used and/or analysed during the current study are available from the corresponding author on reasonable request.

## Abbreviations

AUC: Area under the receiver operating characteristic curve
CT: Cortical thickness
DHI: Dizziness Handicap Inventory
FDR: False discovery rate
FWHM: Full-width at half maximum
GAD-7: Generalized Anxiety Disorder-7
GM: Gray matter
HCs: Healthy controls
HIT-6: Headache Impact Test-6
MIDAS: Migraine Disability Assessment Scale
MNI: Montreal Neurologic Institute
*n*: Number of subjects
PFC: Prefrontal cortex
*p*_FDR_: FDR-corrected *p*
PHQ-9: Patient Health Questionnaire-9
SA: Surface area
SBM: Surface-based morphometry
SFG: Superior frontal gyrus
SPL: Superior parietal lobule
SVM: Support vector machine
VAS: Visual Analog Scale
VBM: Voxel-based morphometry
VM: Vestibular migraine
